# Self-Assembled Gold Nanoparticles as Reusable SERS Substrates for Polyphenolic Compound Detection

**DOI:** 10.3390/ijms252312785

**Published:** 2024-11-28

**Authors:** Arina Pavlova, Ksenia Maleeva, Ivan V. Moskalenko, Vadim Belyaev, Mikhail V. Zhukov, Demid Kirilenko, Kirill V. Bogdanov, Evgeny Smirnov

**Affiliations:** 1 Infochemistry Scientific Center, ITMO University, Lomonosova Str. 9, 191002 St. Petersburg, Russia; 2 International Research and Educational Center for Physics of Nanostructures, ITMO University, Birzhevaya Liniya, 14, 197101 St. Petersburg, Russia

**Keywords:** SERS, gold nanoparticles, self-assembly, liquid–liquid interface, chlorogenic acid, density functional theory

## Abstract

Natural polyphenolic compounds play a pivotal role in biological processes and exhibit notable antioxidant activity. Among these compounds, chlorogenic acid stands out as one of the most widespread and important polyphenols. The accurate detection of chlorogenic acid is crucial for ensuring the quality and classification of the raw materials used in its extraction, as well as the final products in the food, pharmaceutical, and cosmetics industries that contain this bioactive compound. Raman spectroscopy emerges as a powerful analytical tool, particularly in field applications, due to its versatility and sensitivity, offering both qualitative and quantitative analyses. By using the self-assembly of gold nanoparticles at liquid–liquid interfaces and the developed “aqua-print” process, we propose a facile and inexpensive route to fabricate enhanced substrates for surface-enhanced Raman spectroscopy with high reproducibility. To ensure substrate reliability and accurate molecule detection in SERS experiments, a benchmarking procedure was developed. This process involved the use of non-resonant rhodamine 6G dye in the absence of charge transfer and was applied to all synthesized nanoparticles and fabricated substrates. The latter revealed the highest enhancement factor of 4 × 10^4^ for 72 nm gold nanoparticles among nanoparticle diameters ranging from 14 to 99 nm. Furthermore, the enhanced substrate was implemented in the detection of chlorogenic acid with a concentration range from 10 μM to 350 μM, demonstrating high accuracy (R^2^ > 99%). Raman mapping was employed to validate the good uniformity of the signal (the standard deviation was below 15%). The findings of this study were also supported by DFT calculations of the theoretical Raman spectra, demonstrating the formation of the chlorogenic acid dimer. The proposed method is strategically important for the development of the class of in-field methods to detect polyphenolic compounds in raw materials such as plants, extracted plant proteins, and polyphenolic compounds.

## 1. Introduction

Polyphenolic compounds are widespread in nature and valued for their diverse biological activities [1]. One of the most prominent polyphenols is chlorogenic acid (CGA), found in coffee beans [2], sunflower seeds [3], blueberry leaves [4], chicory roots [5], sweet potato roots [6], and other plants. CGA has beneficial bioactivities, namely, antioxidant and DNA-protective properties [7] and anti-inflammatory properties [8], leading to anti-cancer, anti-aging, and even anti-osteoporosis effects [9,10,11,12,13]. Besides this, CGA has the capacity to inhibit some ferments such as α-glucosidase [14], making it widely used as a dietary supplement.

However, as an ester of quinic and caffeic acids, CGA has plenty of isomers due to variations in the binding locations of these acids [1]. It is difficult to analyze CGA compounds from natural raw materials with conventional analytical techniques due to their structural complexity and low abundance. Therefore, it is essential to develop a simple, rapid, and robust method to determine the CGA content in food and pharmaceutical products.

In recent years, Raman spectroscopy has become one of the most promising and rapidly developing analytical methods, allowing both qualitative and quantitative analyses of various analyte molecules [15,16]. A unique property of Raman spectroscopy (RS) is its ability to identify substances in the “fingerprint” region (typically between 300 and 1900 cm^−1^) [15]. This allows for the determination of individual compounds, especially their isomers [17], in objects of complex composition. In particular, surface-enhanced Raman spectroscopy (SERS), developed in 1974 by Fleischmann and colleagues [18], has played a pivotal role in the fast development of Raman spectroscopy as an analytical tool. It has become widespread in entirely different fields, including biomolecule detection [19], bacterium identification [20,21], agriculture [22], food chemistry [23], pharmaceuticals [24], forensics [25,26], ecology [27,28], peptide [29] and volatile [30] detection, and many others.

The origin of the surface enhancement phenomenon in Raman spectroscopy lies in a combination of electromagnetic (associated with localized surface plasmon resonance, or LSPR) and chemical (associated with charge-transfer reactions) mechanisms [31]. Thus, the enhancement factor (EF) in SERS is usually about 10^4^–10^6^ relative to Raman spectra without enhancement. The chemical mechanism makes it possible to achieve an EF of up to 10^6^–10^8^, which is one–two orders of magnitude higher than that for pure electromagnetic mechanisms.

Recently, Liz-Marzán and colleagues formalized 10 challenges that need to be accomplished to make surface-enhanced Raman spectroscopy a fully successful technique, both in research and on the market [15]. Among them are, on the one hand, the development of efficient, inexpensive, reproducible, and reusable substrates and, on the other hand, proof of SERS performance using non-resonant molecules in the absence of charge-transfer resonances.

One of the promising directions for fabricating enhanced substrates is the self-assembly of plasmonic nanoparticles (NPs) at the phase interface [32,33,34,35,36] or in the polymer matrix [37,38,39]. Assembly at the liquid–liquid interface not only allows for arranging particles in a close-packed structure [32,40] but also shortens the distance between separate nanoparticles, leading to uniform and dense hotspots, higher sensitivity, and higher enhancement factors [37,41].

Gold and silver are usually used as plasmonic metals to fabricate such substrates. However, gold nanoparticles (AuNPs) possess relatively better stability to surface reactions and oxidation than silver, as recently reviewed by Kang and co-workers [42]. Thus, SERS substrates based on gold nanoparticles are becoming increasingly popular as sensors for substances of various natures, ranging from classical model molecules such as rhodamine 6G (R6G) [43] to polycyclic aromatic hydrocarbons [28,44].

Additionally, R6G is a good reporter or model molecule for benchmarking SERS substrates, as it possesses low absorbance and, thus, negligible fluorescence in the red part of the spectrum; also, it can be washed off the gold surface if necessary [29].

Predicting Raman spectra with density functional theory (DFT) allows for obtaining information about the molecular structure, chemical bonds, interactions, and physical properties of materials [45]. This approach can be used to analyze different types of materials, from organic compounds to nanomaterials and biomolecules [46,47,48,49]. For compounds such as polyphenols that exhibit intramolecular hydrogen bonds [50], the PBE family of methods has proven to be the most accurate.

To the best knowledge of the authors, only a few recent reports aimed to detect chlorogenic acid by Raman spectroscopy. In [50], the authors discussed the Raman spectra of various regioisomeric forms of monoacyl and diacyl chlorogenic acids, examined both experimentally and theoretically using DFT calculations (B3LYP/6-311G(d,p) level). This study provided valuable insights into the Raman scattering spectral features of CGA isomers. Another relevant work reported the successful detection of CGA in stevia leaves, coffee beans, and other plant samples. The authors utilized Ag-Cu_2_O nanoparticle substrates and achieved a limit of detection for 3-CQA as low as 0.13 μg/mL, corresponding to a molar concentration of 3.67 × 10⁻⁶ M [51]. Furthermore, a more recent study in *Chemosensors* [52] reported the detection of CGA in coffee using standard Raman spectroscopy with a 785 nm laser. However, the authors used classical Raman spectroscopy (not SERS), resulting in a higher minimal detectable concentration of 123 mg/L (equivalent to 0.34 mM or 340 μM).

Therefore, this work presents a comprehensive study of enhanced SERS substrates based on self-assembled nanoparticles with a full physicochemical characterization. Specifically, our focus lies in two key aspects of application-oriented research on nanomaterials: the fabrication of reliable substrates and the benchmarking of these substrates using non-resonant molecules, with further practical applications to detect polyphenols such as chlorogenic acid. The substrates are fabricated by the self-assembly of gold nanoparticles at the liquid–liquid interface of water and dichloroethane and transferred to a solid substrate by the “aqua-print” process. Further, these substrates are benchmarked with non-resonant reporter molecules, such as rhodamine 6G, to demonstrate their reliability. Besides this, we demonstrate the contemporary advancements in the application of the substrates to polyphenolic compound detection via SERS on an example of CGA, as accurate detection of this bioactive compound is crucial for many industrial applications, such as in foods, pharmaceuticals, and cosmetics. Importantly, SERS measurements of both R6G and CGA are supported by Raman mapping to prove SERS performance and to demonstrate the good uniformity of the signal.

## 2. Results and Discussion

### 2.1. Characterization of Synthesized AuNP Colloids

**Figure 1:**
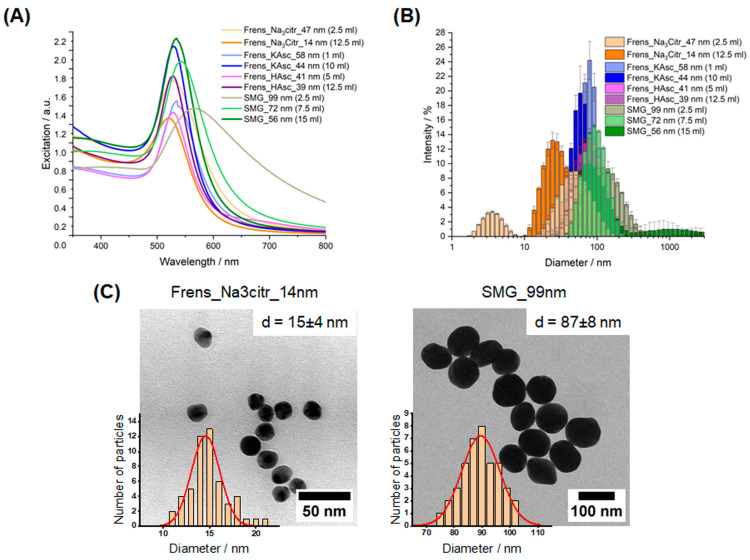
Characterization of synthesized gold nanoparticles. (**A**) UV-Vis absorbance spectra, (**B**) particle size distributions and ζ-potentials, and (**C**) TEM images of synthesized AuNPs of maximal and minimal diameters. Insets: Bar diagrams of particle size distributions based on TEM image data processing (the red curve is the Gaussian fit). The labeling of the samples should be read as the following: *Frens* = nanoparticles synthesized by the Frens–Turkevich method using one reductant agent: *Na_3_Citr* = sodium citrate, *KAsc* = potassium ascorbate, or *HAsc* = ascorbic acid; *SMG* = nanoparticles synthesized by Park’s method.

To find the optimal particle diameter to obtain the highest possible enhancement factor for SERS substrates, gold nanoparticles were synthesized using two distinct methods, namely, the reduction of HAuCl_4_ (commonly referred to as the Frens–Turkevich method) and seed-mediated growth (referred to as Park’s method). A comprehensive characterization of the AuNP colloids was conducted to access their particle diameter and concentration, as well as to estimate the overall colloid stability, which is crucial for precisely tuning the AuNP packing of the film at the liquid–liquid interface. UV-Vis spectroscopy, dynamic light scattering (DLS), and transmission electron microscopy (TEM) were employed to analyze the AuNP colloids (Figure 1). The detailed UV-Vis spectra and particle size distributions for each synthetic procedure are given in the Supplementary Information, Section S3.

Figure 1A depicts the typical absorbance spectra of the obtained AuNP solutions. As anticipated, the position of the absorption peak shifts in response to changes in the volume of reducing agent added during the synthesis stage, indicating changes in the mean particle diameter. For instance, for nanoparticles synthesized by the reduction of trisodium citrate, a drop in the volume of the reducing agent from 12.5 mL to 2.5 mL entails the maximum absorption wavelength changing from 521 nm to 528 nm (orange curves in Figure 1A and Figure S3-1 in the Supplementary Information).

Similarly, with a decrease in the added volume of potassium ascorbate from 10 mL to 1 mL, the absorption peak shifts from 527 nm to 535 nm (blue curves in Figure 1A and Figure S3-2 in the Supplementary Information). Nevertheless, replacing the reducing agent with ascorbic acid and varying the added volume from 12.5 mL to 5 mL leads to only a slight variation in the maximum absorption wavelength, from 527 nm to 528 nm (purple curves in Figure 1A and Figure S3-3 in the Supplementary Information).

For particles synthesized by the seed-mediated growth method, the maximum absorption wavelength steadily increases from 534 nm to 567 nm with the volume of seed nanoparticles (green curves in Figure 1A and Figure S3-4 in the Supplementary Information). These seed nanoparticles were previously synthesized by the Frens–Turkevich method; they were roughly 22 nm in diameter, and the volume added to the growth solution was varied from 15 mL to 2.5 mL.

To determine the diameters and concentrations of gold nanoparticles from the UV-Vis spectra, an approach based on the Haiss methodology [53] was applied from the previously published program [54]. The procedure described above led to gold nanoparticles in a broad range of diameters, namely, from 14 to 99 nm. In particular, AuNPs synthesized with sodium citrate had diameters ranging from 14 nm to 44 nm; those with ascorbic acid, from 39 to 47 nm; those with potassium ascorbate, from 41 to 58 nm; and those synthesized by seed-mediated growth, from 56 nm to 99 nm.

The findings described above were also confirmed by a dynamic light scattering investigation (Figure 1B), and the stability of the prepared colloids was estimated. Detailed data on each synthetic approach are presented in the Supplementary Information, Section S3. The colloids were stable, as the average ζ-potential was in the range of −25 ÷ −30 mV, which is above the stability threshold. Nevertheless, potassium ascorbate and ascorbic acid were worse at stabilizing nanoparticles than trisodium citrate, so the corresponding samples possessed ζ-potential values closer to −25 mV. Despite their lower stability as a colloid, their lifetime was sufficient for the fabricating assemblies at the liquid–liquid interface and then the enhanced substrates. The obtained results for all synthesized samples are summarized in Table S3 in the Supplementary Information.

In addition, a TEM investigation of the synthesized gold nanoparticles was conducted to access the particle morphology (Figure 1C and Figure S4 in the Supplementary Information). In the case of the classical Frens–Turkevich approach with trisodium citrate, the gold nanoparticles were mainly spherical. However, substituting sodium citrate with ascorbic acid led to nanoparticles with greater diameters and the appearance of AuNPs with irregular shapes. A further change to potassium ascorbate led to the formation of triangular particles. In contrast, AuNPs synthesized by seed-mediated growth possessed high sphericity compared to those from other methods.

### 2.2. Characterization of SERS Substrate Morphology

Enhanced substrates were prepared in accordance with the procedure described above for all synthesized gold nanoparticles to select the one with the highest EF. As the packing and nanoparticle arrangement are crucial for the maximum enhancement, the obtained film morphology was characterized.

**Figure 2:**
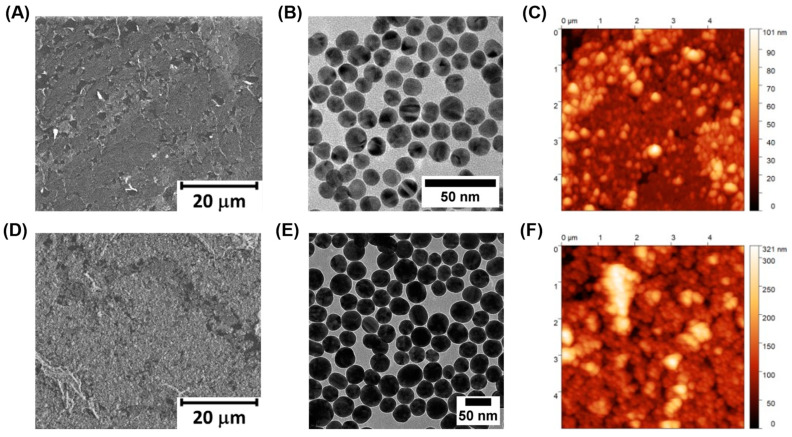
Morphological characterization of the fabricated enhanced substrates by SEM (**A**,**D**), TEM (**B**,**E**), and AFM (**C**,**F**). Silicon substrates coated with 14 nm AuNPs (**A**–**C**) and 72 nm nanoparticles (**D**–**F**).

The uniformity of the films deposited on silicon substrates using the “aqua-print” method was investigated by scanning electron microscopy (SEM) with energy-dispersive X-ray spectroscopy (EDX) microanalysis, transmission electron microscopy (TEM), and atomic force microscopy (AFM); the results are depicted in Figure 2A,D, Figure 2B,E, and Figure 2C,D, respectively.

SEM images at low magnification demonstrated a reasonably uniform deposition of gold nanoparticles over a large surface area, regardless of the nanoparticle diameter (Figure 2A,D represent 14 nm and 72 nm nanoparticles). Additionally, to demonstrate the uniformity of substrate surface coverage, EDX mapping was carried out (Figure S6 in the Supplementary Information). The EDX map of silicon, based on its Kα line, shows the flat background; at the same time, the EDX map of gold, based on its Mα line, demonstrates features such as tiny cracks and pinholes, which are also present in the SE image. The presence of sulfur is negligible according to its EDX mapping.

Even in regions where dark areas indicative of uncovered silicon substrate are observed, the size of these defective areas (typically less than 0.5 μm in the lateral dimension) is substantially smaller (5 to 10 times) than the diameter of a regular laser spot of a Raman spectrometer (typically 3–4 μm). Therefore, the observed defective areas minimally interfere with the applications of the fabricated enhanced substrates.

The TEM images in Figure 2B,E reveal a close-packed arrangement of nanoparticles with some nano-defects, likely resulting from the transfer of the film to a TEM grid. Simultaneously, the AFM images presented in Figure 2C,F demonstrate the uniform coating of the substrate by gold nanoparticles deposited on the silicon substrate.

Thus, a densely packed spherical gold nanoparticle film can be observed, with some areas exhibiting hexagonal packing of the spheres. Most of the coating for both the nanoparticle diameters has a monolayer structure, which is consistent with the experimental design. Overall, the comprehensive characterization confirms the uniformity and structural integrity of the fabricated enhanced substrates for SERS applications.

### 2.3. Substrate Benchmarking by Rhodamine 6G Detection

**Figure 3:**
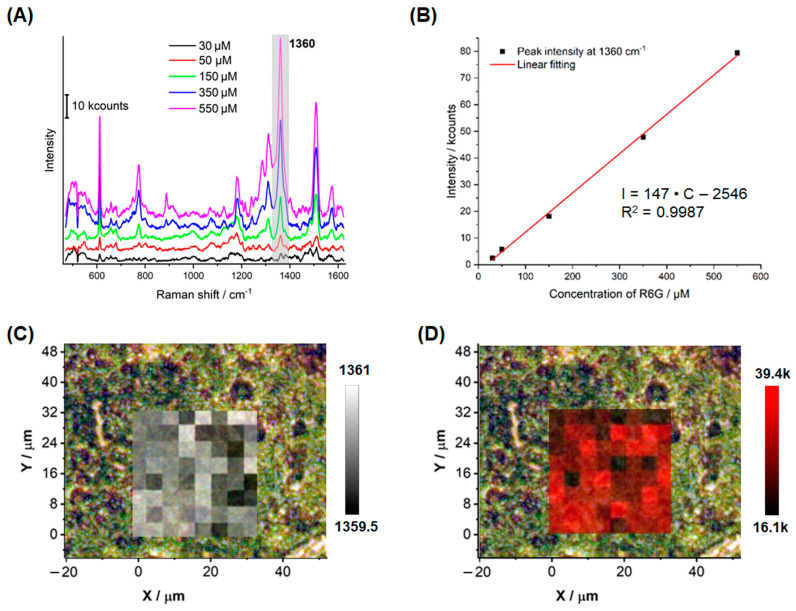
Benchmarking the enhanced substrate made of 14 nm AuNPs with the non-resonant reporter molecule R6G at varying concentrations (the grey area marks out the peak used for plotting panel B). (**A**) Recorded surface-enhanced Raman spectra of R6G. (**B**) Calibration curve demonstrating the linear dependence of the Raman scattering of R6G at a selected wavenumber of 1360 cm^−1^. (**C**,**D**) Mapping of the enhanced substrate at an R6G concentration of 150 μM: (**C**) peak position map and (**D**) peak intensity map at 1360 cm^−1^.

We further investigated the enhanced properties of all the prepared nanofilms transferred onto a silicon substrate using a non-resonant reporter molecule, rhodamine 6G (R6G). A detailed methodology for measuring Raman spectra is outlined in the Materials and Methods Section. Briefly, an ethanol solution of R6G dye was added to the enhanced substrate and dried, followed by Raman spectra measurement. Three distinct spots on the substrate surface were analyzed, ensuring robust data collection (Figure 3). The Raman spectra for the pure AuNP-covered substrates are provided in Figure S6 of the Supplementary Information.

For nanoparticles with a diameter of 14 nm, the acquired data exhibited a gradual increase in Raman scattering intensity upon the addition of R6G dye (Figure 3A). The spectra revealed characteristic peaks corresponding to rhodamine 6G, consistent with the positions observed in previously published articles [55,56,57]. This consistency was further confirmed by comparing DFT-calculated and experimental R6G Raman spectra (Figure S7 in the Supplementary Information). The scaling factor for the Raman shift scale was found to be 0.96, which is in line with the values reported in the existing literature [58,59]. The analytical enhancement factor was calculated to be 2.3 × 10^3^ according to Equation (1).

The full profile analysis of the present Raman spectra and the analysis of the Raman scattering intensities revealed the strong linear correlation between the signal and concentration. Such a plot for one of the main R6G peaks at 1360 cm^−1^ is presented in Figure 3B. Notably, a high R^2^ factor of 0.9987 was calculated across more than an order of magnitude in the concentration range, demonstrating the accuracy and reliability of our measurements. The limit of detection (LOD), determined through linearization of the experimental data in accordance with a previously published procedure [29,60], was calculated as 67 μM.

Further, to prove the effectiveness and uniformity of the enhancement properties of the substrates, as well as to validate the reliability of the fabricated SERS substrate, Raman signal mapping was conducted at an R6G concentration of 150 μM (the middle of the studied concentration range). Two primary parameters were assessed: the position of the most intense peak (Figure 3C) and the peak intensity (Figure 3D).

As can be clearly seen in Figure 3C, the peak position varies minimally from one spot to another, with variations not exceeding 2 cm^−1^. The peak intensities have a standard deviation of ±19% (5200) from the mean value (ca. 26,100).

**Figure 4:**
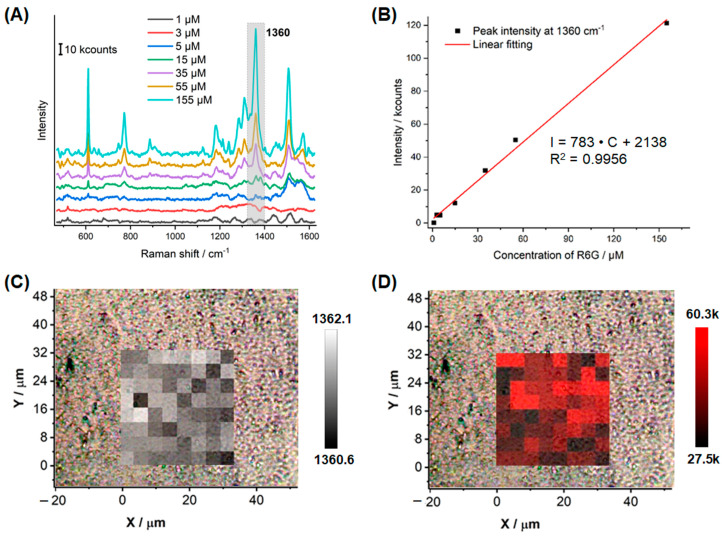
Benchmarking the enhanced substrate made of 72 nm AuNPs with the non-resonant reporter molecule R6G at varying concentrations. (**A**) Recorded surface-enhanced Raman spectra of R6G (the grey area marks out the peak used for plotting panel B). (**B**) Calibration curve demonstrating the linear dependence of Raman scattering of R6G at a selected wavenumber of 1360 cm^−1^. (**C**,**D**) Mapping of the enhanced substrate at an R6G concentration of 150 μM: (**C**) peak position map and (**D**) peak intensity map at 1360 cm^−1^.

Similarly, for larger (72 nm) gold nanoparticles, Raman benchmarking was conducted (Figure 4). The Raman signal exhibited a gradual increase with the concentration of added R6G dye (Figure 4A), leading to a linear concentration-dependent signal (Figure 4B) with an R^2^ factor of 0.9955. The AEF, calculated from the Raman spectra, was determined to be 4 × 10^4^, indicating significant electromagnetic non-resonant enhancement according to Ref. [31].

Likewise, for smaller nanoparticles, the fitted position of the peak negligibly shifts around 1361 cm^−1^ (Figure 4C), while the peak intensities have a standard deviation of ±20% (8000) of the mean value (ca. 39,500) (Figure 4D). The calculated LOD is 19 μM. In both cases, the most pronounced enhancements in signal intensities can be associated with macroscopic defects in the nanoparticle film, like pinholes, cracks, or scratches.

**Figure 5:**
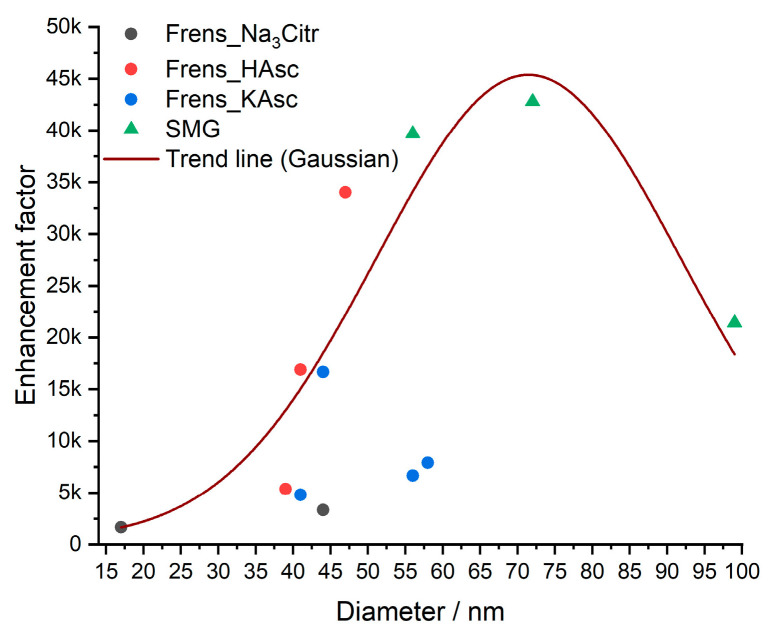
Dependence of the analytical enhancement factor on the diameter of AuNPs synthesized by different methods. A Gaussian curve was used as the trend line.

Similar procedures were employed for all fabricated enhanced substrates. In the majority of cases, the calculated R^2^ factor exceeded 98%, affirming the robustness of the measurements. Therefore, the AEF was chosen as the most expressive characteristic and informative metric for substrate selection in the detection of polyphenolic compounds. Figure 5 presents all the obtained data on the AEF.

The relationship between the AEF and the diameter of the nanoparticles used to fabricate the enhanced substrate exhibits a bell-shaped (Gaussian) curve, with the maximum AEF occurring for nanoparticles ranging from 56 to 72 nm in diameter, synthesized via seed-mediated growth. This corroborates previously published results for cubes [61] and spheres [62]. The bell or Gaussian shape arises as an interplay of localized surface plasmon resonance (LSPR), electromagnetic field enhancement, and light scattering/absorption. For small particles (smaller than 30 nm in diameter), the resonance is weaker because of smaller cross-sections for light interaction, whereas for big particles (larger than 90 nm in diameter), the electromagnetic enhancement diminishes because of increased radiative damping and a shift in the resonance frequency away from the optimal range for the excitation light, along with increased scattering over absorption. Consequently, the enhanced substrate made of 72 nm AuNPs was selected for the further detection of chlorogenic acid, serving as an example of phenolic compounds.

### 2.4. Application of the Enhanced Substrate to Chlorogenic Acid Detection

**Figure 6:**
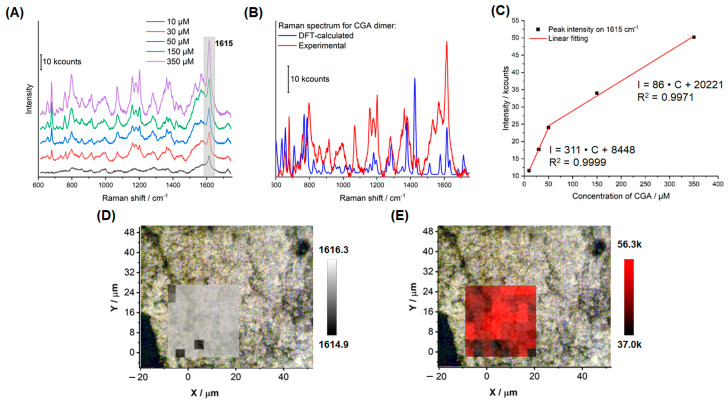
Detection of CGA dimer on the enhanced substrate made of 72 nm AuNPs by Raman spectroscopy. (**A**) Experimental spectra on SERS substrates for different concentrations of CGA dimer (the grey area marks out the peak used for plotting panel C). (**B**) Comparison of the DFT-calculated Raman spectrum with the experimental one. (**C**) Calibration curve demonstrating the linear dependence of Raman scattering of the CGA dimer at 1615 cm^−1^. (**D**,**E**) Mapping of the enhanced substrate at a CGA concentration of 50 μM: (**C**) peak position map and (**D**) peak intensity map at 1615 cm^−1^.

Finally, to demonstrate the applicability of the enhanced substrate in the detection of complex molecules, the Raman spectra of CGA were recorded. The deposition procedure on the substrate was similar to the one described above for R6G dye. However, detecting Raman signals proved challenging due to the significant fluorescence emanating from the silicon substrate with deposited AuNPs and CGA. Nevertheless, a small amount of ammonia was added to the CGA to facilitate its conversion into a dimeric form. This conversion was evidenced by a noticeable change in color of the most concentrated solution (10^−4^ M) from colorless to slightly yellowish, indicating a successful transition from the monomer to the dimer state. Subsequent experiments were conducted with the CGA dimer (Figure 6) and to confirm that the obtained results belonged to the CGA dimer.

The recorded Raman spectra exhibit a consistent increase in intensity with the addition of CGA dimers at different concentrations of CGA, ranging from 10 to 350 μM (Figure 6A). These data reveal several distinct peaks attributed to the CGA dimer. The theoretical Raman spectrum for the CGA dimer was calculated using DFT and compared with the experimental spectrum (Figure 6B). All main peaks identified from the full profile analysis are listed in Table S8 in the Supplementary Information, along with their respective vibration types and corresponding bonds. Additionally, Table S8 includes peak data retrieved from DFT calculations, both scaled and non-scaled. As in the procedure described above for R6G dye, a scaling factor of 0.96 was applied to the theoretical Raman spectrum. Remarkably, the theoretical Raman spectrum aligns closely with the experimental results, underscoring the fidelity of the DFT calculation results.

The peak intensities derived from the full profile analysis for the band at 1615 cm^−1^ exhibit two distinct linear dependencies, with a noticeable bending point in the middle of the concentration range (Figure 6C). This bend suggests the possibility of surface saturation with molecules, similar to that in Ref. [29], or potentially indicates self-quenching of the signal. The LOD from the first linear equation for lower CGA loadings was estimated as 59μM. The calculated value is consistent with those for recently developed electrochemical sensors [63].

Similarly to the benchmarking of substrates with R6G, mapping of the Raman peak position and signal intensity from the CGA dimer was carried out to validate the enhancing properties of the substrate (Figure 6D,E). The peak position varies negligibly among the spots except for at 2–3 points, where the drop can be associated with pinholes in the gold nanoparticle layer (Figure 6D). The peak intensity at 1615 cm^−1^ reveals a relatively uniform spatial distribution of the Raman signal (Figure 6E). Both slight shifts in the peak position and drops in intensity occur only at defects and pinholes; therefore, if those spots are excluded, the peak intensities have a standard deviation of ± 10% (4500) from the mean intensity value (ca. 45,200).

## 3. Materials and Methods

### 3.1. Reagents and Chemicals

Solutions of hydrogen tetrachloroaurate (III) (HAuCl_4_·3H_2_O, 23.5–23.8 w% Au) were purchased from Aladdin (Shanghai, China). Trisodium citrate (Na_3_C_6_H_5_O_7_·2H_2_O, 98%), potassium ascorbate (KC_6_H_7_O_6_, 98%), ascorbic acid (C_6_H_8_O_6_, 99%), ammonia solution (23 w%), silver nitrate (AgNO_3_, 99.9%), and ethanol (C_2_H_6_O, 96%) were received from Macklin (Shanghai, China). Rhodamine 6G (R6G, C_28_H_31_ClN_2_O_3_, 98%), chlorogenic acid (CGA, C_16_H_18_O_9_, 99%), dichloroethane (DCE, C_2_H_4_Cl_2_, >99%), and hexane (C_6_H_14_, >99%) were ordered from Aldrich (Milwaukee, WI, USA). Silicon substrates were purchased from Sigma-Aldrich (Burlington, MA, USA).

### 3.2. Synthesis of AuNPs

**Scheme 1:**
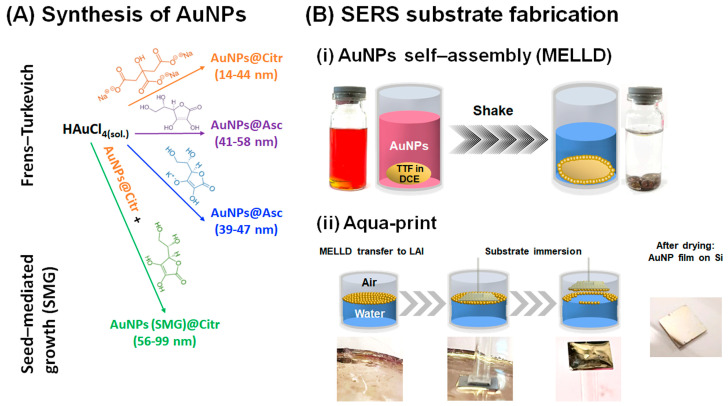
Schematic of AuNP synthesis and SERS substrate preparation procedure. (**A**) Synthesis of AuNPs with different methods: Frens–Turkevich with trisodium citrate, ascorbic acid, or potassium ascorbate; seed-mediated growth from smallest seed particle obtained by Ferns–Turkevich method. (**B**) Enhanced substrate fabrication: (**i**) self-assembly of AuNPs into nanofilm using liquid–liquid interface (MELLDs), (**ii**) transfer of nanofilm to solid substrate (silicon) with aqua-print technology. Photos demonstrate visual appearance of MELLD and nanofilm on substrate.

Gold nanoparticles were synthesized using two different methods to access a broad range of nanoparticle diameters (Scheme 1). The first classical synthetic approach was carried out according to the works of Frens and Turkevich [64,65] with modifications discussed in Ref. [54]. This method involves the reduction of hydrogen tetrachloroaurate (III) using one of the following reducing agents: trisodium citrate, potassium ascorbate, or ascorbic acid. The second approach was “seed nanoparticle growth”, which was based on the work of Y. Park and S. Park [66]. All necessary details are given in Section S2 of the Supplementary Information.

### 3.3. Preparation of Enhanced Substrates

To prepare enhanced substrates for SERS, a two-step process was adapted from Reference [33]. In the first step, the self-assembly of nanoparticles was carried out for all obtained samples at the interface between two immiscible liquids, in accordance with the previously published procedure by our group [32,54]. All necessary details are given in Section S2 of the Supplementary Information.

In the second step, AuNP film transfer was carried out using the “aqua-print” method, which was redesigned from the previously published “lift-on” method [67]. To do so, the as-prepared self-assembled films were transferred to a water–organic interface (a mixture of hexane with 1,2-dichloroethane in a ratio of 2 to 1) in a beaker; then, the organic layer was carefully removed. Finally, the silicon substrate was immersed into a film of nanoparticles; no special installation is required for this.

### 3.4. Characterization of Aqueous Colloidal AuNP Solutions

The synthesized colloids of AuNPs were characterized by UV-Vis absorption spectroscopy with a UV-1800 spectrophotometer (Shimadzu, Kyoto, Japan) with a 10 mm quartz cuvette and by dynamic light scattering (DLS) with ζ-potential measurements using a Zetasizer Nano (Malvern, UK). The average particle diameter and concentration were calculated from the absorption spectra according to previously published works [53,54]. The sample morphology was investigated by transmission electron microscopy (TEM), using a JEM-2100F microscope (Jeol, Tokyo, Japan).

### 3.5. Characterization of the Morphology of Enhanced Substrates

All substrates were cleaned before deposition using a Femto low-pressure plasma system (Diener, Ebhausen, Germany) for a duration of 5 min.

The uniformity of the AuNPs deposited on silicon substrates using the method described above was investigated using scanning electron (SEM) and atomic force (AFM) microscopy. SEM images were obtained using a Quanta Inspect microscope (FEI Company, Eindhoven, The Netherlands) equipped with a thermionic electron source operating at an accelerating voltage of 20 kV and with a secondary electron detector.

The elemental mapping using Energy-Dispersive X-ray spectroscopy (EDX) was carried out using the microscope mentioned above at 20 kV. The images were taken with accumulated X-rays for 8 cycles at a resolution of 512 × 400 pixels for each element, the dead time was 30–40%, and the spot of the e-beam was 4.5. The elements Au, Si, and S were selected as the regions of interest; the elements are highlighted in different colors.

AFM images were obtained using an NTEGRA Prima microscope (NT-MDT, Zelenograd, Russia) equipped with an NS15 cantilever (NT-MDT, Zelenograd, Russia); the typical radius of the tip was 8 nm.

### 3.6. Raman Spectra on the Enhanced Substrates

All Raman spectra were recorded with an InVia Raman microscope (Renishaw, Wotton-under-Edge, UK) using a He-Ne laser with a wavelength of 633 nm. To obtain the spectra, the typical parameters were the following: 5 or 10 s for a single scan, 10 accumulations, with a delivered power of approximately 0.5 mW on the sample surface.

The typical procedure involved the deposition of a 10 μL droplet of R6G dye solution on the enhanced substrates with a surface area of −1 cm^2^ and subsequent drying. The dye concentrations ranged from 10^−7^ M to 10^−5^ M in ethanol to intensify the drying process. After the droplet had completely dried, the Raman spectra were recorded. Typically, the spectra were recorded from three different spots on the substrate surface and analyzed in the WiRE (version 5.3) package to extract the peak intensities; further, the mean intensity and standard deviation were calculated. A similar procedure was applied for the CGA solution, except that the pH was tuned to ca. 10 with ammonia solution in order to promote dimer formation.

To calculate the enhancement factor (EF), the Raman spectrum of pure rhodamine 6G was recorded after applying a 4 × 10^−^^2^ M dye solution with a volume of 10 μL to the surface of a pristine silicon substrate. The analytical EF, as the most practical one to compare, was calculated with the following equation [68]:

(1) AEF=ISERSCSERSIRSCRS,

where *C_SERS_* and *C_RS_* are the analyte concentrations with and without enhancement, and *I_SERS_* and *I_RS_* are the respective signal intensities.

For mapping experiments, a grid of 8 × 8 points was scanned with a step size of 3 µm between each point. The spatially resolved Raman data allowed for the investigation of sample heterogeneity and the identification of localized SERS hotspots. All maps were treated and plotted with WiRE software (version 5.3). The detailed procedure for map acquisition is presented in our previous publication [29].

Substrate cleaning before and after the measurement was performed with a low-pressure plasma system, Femto (Diener, Ebhausen, Germany), for a duration of 5 min. Additionally, this allowed the formation of an ultrathin layer of gold oxide and improved performance in SERS [30].

### 3.7. Computation Details

The full geometric optimization and Raman spectrum for the structure of CGA were obtained at the PBE0-D3/def2-TZVP level of theory with the help of the Orca 5.0.3 program package [69]. The RIJCOSX approximation [70,71] utilizing the def2/J auxiliary basis set and spin-restricted approximation were employed. The solvent effects were considered by the CPCM model [72]. The reasonable assumption is that, because the substrates were used at ambient room humidity (roughly 45–55%) without an additional drying procedure such as desiccation or vacuum drying, a tiny layer of water was present at the surface.

The Hessian matrices were calculated for all optimized model structures to determine the location of the correct stationary points on the potential energy surfaces (no imaginary frequencies were found in any of the cases) and to estimate the thermodynamic properties (Gibbs free energy) for all model systems at 298.15 K and 1 atm. The Cartesian atomic coordinates of the structures are presented in the Supplementary Information. The calculated Hessian files were processed in vibAnalysis 1.2.2 [73,74].

## 4. Conclusions

To sum up, in this study, we presented an effective, cost-efficient, and high-throughput method for fabricating enhanced substrates for SERS. The approach relies on the self-assembly of gold nanoparticles at the liquid–liquid interface, followed by transfer to a solid substrate by the “aqua-print” technique. The latter allowed us to fabricate enhanced substrates made of arranged gold nanoparticles in a wide range of nanoparticle diameters, from 14 to 99 nm. These substrates were benchmarked to assess their enhancing characteristics with a non-resonant molecule, R6G, in the absence of charge transfer. Across all considered samples, a linear calibration curve spanning more than an order of magnitude in the R6G concentration was achieved. The LODs for most samples were at the level of ca. 60 μM or below. Furthermore, these substrates demonstrated high spatial uniformity of their Raman scattering signals, as confirmed by Raman mapping, along with analytical enhancement factors of up to ca. 4 × 10^4^, affirming their efficacy in SERS experiments.

Additionally, we demonstrated advancements in the application of these substrates to the SERS detection of polyphenolic compounds, such as CGA. The Raman spectrum of CGA was validated through DFT calculations. With the excellent linearity of the Raman signal versus the concentration and an LOD of 19 μM, the presented data underscore the high applicability of the fabricated substrates for the quantitative detection of polyphenolic compounds. Importantly, the surface of such a substrate may be easily renewed using oxygen plasma at any time, ensuring their reusability at negligible cost.

Overall, the presented methodology offers highly reproducible fabrication of substrates through self-assembly at the liquid–liquid interface, providing a versatile and cost-effective platform for SERS applications. Moving forward, we intend to apply this method to investigate chlorogenic acid extracts obtained from sunflower cake, further expanding the scope of the research.

## Data Availability

Dataset available on request from the authors.

## Supplementary Materials

The following supporting information can be downloaded at: https://www.mdpi.com/article/10.3390/ijms252312785/s1.

## References

[1] Lu H., Tian Z., Cui Y., Liu Z., Ma X. (2020). Chlorogenic acid: A comprehensive review of the dietary sources, processing effects, bioavailability, beneficial properties, mechanisms of action, and future directions. Compr. Rev. Food Sci. Food Saf..

[2] Loader T.B., Taylor C.G., Zahradka P., Jones P.J. (2017). Chlorogenic acid from coffee beans: Evaluating the evidence for a blood pressure–regulating health claim. Nutr. Rev..

[3] Sosulski F. (1979). Organoleptic and nutritional effects of phenolic compounds on oilseed protein products: A review. J. Am. Oil Chem. Soc..

[4] Harris C.S., Burt A.J., Saleem A., Le P.M., Martineau L.C., Haddad P.S., Bennett S.A.L., Arnason J.T. (2007). A single HPLC-PAD-APCI/MS method for the quantitative comparison of phenolic compounds found in leaf, stem, root and fruit extracts of *Vaccinium angustifolium*. Phytochem. Anal..

[5] Milala J., Grzelak K., Król B., Juśkiewicz J., Zduńczyk Z. (2009). Composition and properties of chicory extracts rich in fructans and polyphenols. Pol. J. Food Nutr. Sci..

[6] Ishiguro K., Yahara S., Yoshimoto M. (2007). Changes in Polyphenolic Content and Radical-Scavenging Activity of Sweetpotato (*Ipomoea batatas*L.) during Storage at Optimal and Low Temperatures. J. Agric. Food Chem..

[7] Xu J.-G., Hu Q.-P., Liu Y. (2012). Antioxidant and DNA-Protective Activities of Chlorogenic Acid Isomers. J. Agric. Food Chem..

[8] Wang H., Zhao Y., Zhang Y., Yang T., Zhao S., Sun N., Tan H., Zhang H., Wang C., Fan H. (2022). Effect of Chlorogenic Acid via Upregulating Resolvin D1 Inhibiting the NF-κB Pathway on Chronic Restraint Stress-Induced Liver Inflammation. J. Agric. Food Chem..

[9] Bisht A., Dickens M., Rutherfurd-Markwick K., Thota R., Mutukumira A.N., Singh H. (2020). Chlorogenic Acid Potentiates the Anti-Inflammatory Activity of Curcumin in LPS-Stimulated THP-1 Cells. Nutrients.

[10] Hu X., Wang M., Pan Y., Xie Y., Han J., Zhang X., Niayale R., He H., Li Q., Zhao T. (2020). Anti-inflammatory Effect of Astragalin and Chlorogenic Acid on Escherichia coli-Induced Inflammation of Sheep Endometrial Epithelium Cells. Front. Vet. Sci..

[11] Liang N., Kitts D.D. (2018). Chlorogenic Acid (CGA) Isomers Alleviate Interleukin 8 (IL-8) Production in Caco-2 Cells by Decreasing Phosphorylation of p38 and Increasing Cell Integrity. Int. J. Mol. Sci..

[12] Wang D., Tian L., Lv H., Pang Z., Li D., Yao Z., Wang S. (2020). Chlorogenic acid prevents acute myocardial infarction in rats by reducing inflammatory damage and oxidative stress. Biomed. Pharmacother..

[13] Kurata R., Adachi M., Yamakawa O., Yoshimoto M. (2006). Growth Suppression of Human Cancer Cells by Polyphenolics from Sweetpotato (*Ipomoea batatas* L.) Leaves. J. Agric. Food Chem..

[14] Abioye R.O., Nwamba O.C., Okagu O.D., Udenigwe C.C. (2023). Synergistic Effect of Acarbose–Chlorogenic Acid on α-Glucosidase Inhibition: Kinetics and Interaction Studies Reveal Mixed-Type Inhibition and Denaturant Effect of Chlorogenic Acid. ACS Food Sci. Technol..

[15] Langer J., Jimenez de Aberasturi D., Aizpurua J., Alvarez-Puebla R.A., Auguié B., Baumberg J.J., Bazan G.C., Bell S.E.J., Boisen A., Brolo A.G. (2020). Present and Future of Surface-Enhanced Raman Scattering. ACS Nano.

[16] Tran V.A., Tran T.T.V., Le V.T., Doan V.D., Vo G.N., Tran V.H., Jeong H., Vo T.T.T. (2024). Advanced nano engineering of surface-enhanced Raman scattering technologies for sensing applications. Appl. Mater. Today.

[17] Gillibert R., Balakrishnan G., Deshoules Q., Tardivel M., Magazzù A., Donato M.G., Maragò O.M., de La Chapelle M.L., Colas F.J., Lagarde F. (2019). Raman Tweezers for Small Microplastics and Nanoplastics Identification in Seawater. Environ. Sci. Technol..

[18] Wang W., Wang W., Liu L., Xu L., Kuang H., Zhu J., Xu C. (2016). Nanoshell-Enhanced Raman Spectroscopy on a Microplate for Staphylococcal Enterotoxin B Sensing. ACS Appl. Mater. Interfaces.

[19] Haidar I., Day A., Martino U., Chevillot-Biraud A., Félidj N., Boubekeur-Lecaque L. (2019). Bottom-up assembly of Au@Ag plasmonic nanocrystals: Issues to be addressed to achieve a good SERS substrate. Appl. Mater. Today.

[20] Leong S.X., Tan E.X., Han X., Luhung I., Aung N.W., Nguyen L.B.T., Tan S.Y., Li H., Phang I.Y., Schuster S. (2023). Surface-Enhanced Raman Scattering-Based Surface Chemotaxonomy: Combining Bacteria Extracellular Matrices and Machine Learning for Rapid and Universal Species Identification. ACS Nano.

[21] De Marchi S., Bodelón G., Vázquez-Iglesias L., Liz-Marzán L.M., Pérez-Juste J., Pastoriza-Santos I. (2018). Surface-enhanced Raman scattering (SERS) imaging of bioactive metabolites in mixed bacterial populations. Appl. Mater. Today.

[22] Yang D., Ying Y. (2011). Applications of Raman Spectroscopy in Agricultural Products and Food Analysis: A Review. Appl. Spectrosc. Rev..

[23] Huang X., Liu Y., Barr J., Song J., He Z., Wang Y., Nie Z., Xiong Y., Chen X. (2018). Controllable self-assembled plasmonic vesicle-based three-dimensional SERS platform for picomolar detection of hydrophobic contaminants. Nanoscale.

[24] Saleh T.A., Al-Shalalfeh M.M., Onawole A.T., Al-Saadi A.A. (2017). Ultra-trace detection of methimazole by surface-enhanced Raman spectroscopy using gold substrate. Vib. Spectrosc..

[25] Izake E.L. (2010). Forensic and homeland security applications of modern portable Raman spectroscopy. Forensic Sci. Int..

[26] Du S., Su M., Jiang Y., Yu F., Xu Y., Lou X., Yu T., Liu H. (2019). Direct Discrimination of Edible Oil Type, Oxidation, and Adulteration by Liquid Interfacial Surface-Enhanced Raman Spectroscopy. ACS Sens..

[27] Nguyen L.B.T., Leong Y.X., Koh C.S.L., Leong S.X., Boong S.K., Sim H.Y.F., Phan-Quang G.C., Phang I.Y., Ling X.Y. (2022). Inducing Ring Complexation for Efficient Capture and Detection of Small Gaseous Molecules Using SERS for Environmental Surveillance. Angew. Chem. Int. Ed..

[28] Montes-García V., Gómez-González B., Martínez-Solís D., Taboada J.M., Jiménez-Otero N., de Uña-Álvarez J., Obelleiro F., García-Río L., Pérez-Juste J., Pastoriza-Santos I. (2017). Pillar [5] arene-Based Supramolecular Plasmonic Thin Films for Label-Free, Quantitative and Multiplex SERS Detection. ACS Appl. Mater. Interfaces.

[29] Grys D.-B., Niihori M., Arul R., Sibug-Torres S.M., Wyatt E.W., de Nijs B., Baumberg J.J. (2023). Controlling Atomic-Scale Restructuring and Cleaning of Gold Nanogap Multilayers for Surface-Enhanced Raman Scattering Sensing. ACS Sens..

[30] Le Ru E.C., Auguié B. (2024). Enhancement Factors: A Central Concept during 50 Years of Surface-Enhanced Raman Spectroscopy. ACS Nano.

[31] Smirnov E., Scanlon M.D., Momotenko D., Vrubel H., Méndez M.A., Brevet P.-F., Girault H.H. (2014). Gold Metal Liquid-Like Droplets. ACS Nano.

[32] Smirnov E. (2018). Perspectives: From Colloidosomes Through SERS to Electrically Driven Marangoni Shutters. Assemblies of Gold Nanoparticles at Liquid-Liquid Interfaces: From Liquid Optics to Electrocatalysis.

[33] Wang Y., Xu X., Fang Y., Yang S., Wang Q., Liu W., Zhang J., Liang D., Zhai W., Qian K. (2024). Self-Assembled Hyperbranched Gold Nanoarrays Decode Serum United Urine Metabolic Fingerprints for Kidney Tumor Diagnosis. ACS Nano.

[34] Tian T., Yi J., Liu Y., Li B., Liu Y., Qiao L., Zhang K., Liu B. (2021). Self-assembled plasmonic nanoarrays for enhanced bacterial identification and discrimination. Biosens. Bioelectron..

[35] Ye Z., Li C., Chen Q., Xu Y., Bell S.E.J. (2021). Self-assembly of colloidal nanoparticles into 2D arrays at water–oil interfaces: Rational construction of stable SERS substrates with accessible enhancing surfaces and tailored plasmonic response. Nanoscale.

[36] Maleeva K.A., Kaliya I.E., Tkach A.P., Babaev A.A., Baranov M.A., Berwick K., Perova T.S., Baranov A.V., Bogdanov K.V. (2022). Formation of Gold Nanoparticle Self-Assembling Films in Various Polymer Matrices for SERS Substrates. Materials.

[37] Maleeva K., Dadadzhanov D., Palekhova A., Kaliya I., Tkach A., Baranov A., Bogdanov K. (2023). SERS substrates based on polymer-protected self-assembled plasmonic films with gold nanoparticles as enhancing element of a microfluidic sensor. Opt. Mater..

[38] Smirnov E., Peljo P., Scanlon M.D., Gumy F., Girault H.H. (2016). Self-healing gold mirrors and filters at liquid–liquid interfaces. Nanoscale.

[39] Smirnov E. (2018). Self-Assembly of Nanoparticles into Gold Metal Liquid-like Droplets (MeLLDs). Assemblies of Gold Nanoparticles at Liquid-Liquid Interfaces: From Liquid Optics to Electrocatalysis.

[40] Li J.F., Tian X.D., Li S.B., Anema J.R., Yang Z.L., Ding Y., Wu Y.F., Zeng Y.M., Chen Q.Z., Ren B. (2012). Surface analysis using shell-isolated nanoparticle-enhanced Raman spectroscopy. Nat. Protoc..

[41] Kang H., Buchman J.T., Rodriguez R.S., Ring H.L., He J., Bantz K.C., Haynes C.L. (2018). Stabilization of Silver and Gold Nanoparticles: Preservation and Improvement of Plasmonic Functionalities. Chem. Rev..

[42] Suganami Y., Oshikiri T., Mitomo H., Sasaki K., Liu Y.-E., Shi X., Matsuo Y., Ijiro K., Misawa H. (2024). Spatially Uniform and Quantitative Surface-Enhanced Raman Scattering under Modal Ultrastrong Coupling Beyond Nanostructure Homogeneity Limits. ACS Nano.

[43] Sánchez-Alvarado A.B., Zhou J., Jin P., Neumann O., Senftle T.P., Nordlander P., Halas N.J. (2023). Combined Surface-Enhanced Raman and Infrared Absorption Spectroscopies for Streamlined Chemical Detection of Polycyclic Aromatic Hydrocarbon-Derived Compounds. ACS Nano.

[44] Qi Z., Akhmetzhanov T., Pavlova A., Smirnov E. (2023). Reusable SERS Substrates Based on Gold Nanoparticles for Peptide Detection. Sensors.

[45] Pagliai M., Osticioli I., Nevin A., Siano S., Cardini G., Schettino V. (2018). DFT calculations of the IR and Raman spectra of anthraquinone dyes and lakes. J. Raman Spectrosc..

[46] Sun S., Brown A. (2014). Simulation of the Resonance Raman Spectrum for Uracil. J. Phys. Chem. A.

[47] Hudson M.R., Allis D.G., Hudson B.S. (2010). The Vibrational Spectrum of Parabanic Acid by Inelastic Neutron Scattering Spectroscopy and Simulation by Solid-State DFT. J. Phys. Chem. A.

[48] Ding Z., Tommasini M., Maestri M. (2017). First-Principles Simulation of Raman Spectra of Adsorbates on Metal Surfaces. ChemPlusChem.

[49] Rodrigues-Oliveira A.F., Ribeiro F.W.M., Cervi G., Correra T.C. (2018). Evaluation of Common Theoretical Methods for Predicting Infrared Multiphotonic Dissociation Vibrational Spectra of Intramolecular Hydrogen-Bonded Ions. ACS Omega.

[50] Eravuchira P.J., El-Abassy R.M., Deshpande S., Matei M.F., Mishra S., Tandon P., Kuhnert N., Materny A. (2012). Raman spectroscopic characterization of different regioisomers of monoacyl and diacyl chlorogenic acid. Vib. Spectrosc..

[51] Caro C., Gámez F., Zaderenko A.P. (2018). Preparation of Surface-Enhanced Raman Scattering Substrates Based on Immobilized Silver-Capped Nanoparticles. J. Spectrosc..

[52] Herdt D., Teumer T., Keck S.P., Kunz T., Schiwek V., Kühnemuth S., Methner F.-J., Rädle M. (2024). Quantitative Analysis of Chlorogenic Acid during Coffee Roasting via Raman Spectroscopy. Chemosensors.

[53] Su J. (2008). A Change Detection Algorithm for Man-made Objects Based on Multi-temporal. Acta Autom. Sin..

[54] Smirnov E. (2018). Self-Assembly of Nanoparticles into Gold Metal Liquid-like Droplets (MeLLDs). Assemblies of Gold Nanoparticles at Liquid-Liquid Interfaces: From Liquid Optics to Electrocatalysis.

[55] Huo C., Han W., Tang W., Duan X. (2021). Stable SERS substrate based on highly reflective metal liquid-like films wrapped hydrogels for direct determination of small molecules in a high protein matrix. Talanta.

[56] de Barros A., Shimizu F.M., de Oliveira C.S., Sigoli F.A., dos Santos D.P., Mazali I.O. (2020). Dynamic Behavior of Surface-Enhanced Raman Spectra for Rhodamine 6G Interacting with Gold Nanorods: Implication for Analyses under Wet versus Dry Conditions. ACS Appl. Nano Mater..

[57] Zhang Y., Zheng J., Guo G., Wang K. (2011). Biosynthesis of gold nanoparticles using chloroplasts. Int. J. Nanomed..

[58] Merrick J.P., Moran D., Radom L. (2007). An Evaluation of Harmonic Vibrational Frequency Scale Factors. J. Phys. Chem. A.

[59] Malik M., Wysokiński R., Zierkiewicz W., Helios K., Michalska D. (2014). Raman and Infrared Spectroscopy, DFT Calculations, and Vibrational Assignment of the Anticancer Agent Picoplatin: Performance of Long-Range Corrected/Hybrid Functionals for a Platinum(II) Complex. J. Phys. Chem. A.

[60] Rukhlyada K.A., Matytcina V.V., Baldina A.A., Volkova O., Kozodaev D.A., Barakova N.V., Orlova O.Y., Smirnov E., Skorb E.V. (2023). Universal Method Based on Layer-by-Layer Assembly for Aptamer-Based Sensors for Small-Molecule Detection. Langmuir.

[61] Lin Y., Zhang Y.-J., Yang W.-M., Dong J.-C., Fan F.-R., Zhao Y., Zhang H., Bodappa N., Tian X.-D., Yang Z.-L. (2019). Size and dimension dependent surface-enhanced Raman scattering properties of well-defined Ag nanocubes. Appl. Mater. Today.

[62] Hong S., Li X. (2013). Optimal Size of Gold Nanoparticles for Surface-Enhanced Raman Spectroscopy under Different Conditions. J. Nanomater..

[63] Alpar N., Yardım Y., Şentürk Z. (2018). Selective and simultaneous determination of total chlorogenic acids, vanillin and caffeine in foods and beverages by adsorptive stripping voltammetry using a cathodically pretreated boron-doped diamond electrode. Sens. Actuators B Chem..

[64] Turkevich J., Stevenson P.C., Hillier J. (1951). A study of the nucleation and growth processes in the synthesis of colloidal gold. Discuss. Faraday Soc..

[65] Frens G., Overbeek J.T.G. (1969). Carey Lea’s colloidal silver. Kolloid Z.U.Z. Polym..

[66] Park Y.-K., Park S. (2008). Directing Close-Packing of Midnanosized Gold Nanoparticles at a Water/Hexane Interface. Chem. Mater..

[67] Song L., Bin Xu B., Cheng Q., Wang X., Luo X., Chen X., Chen T., Huang Y. (2021). Instant interfacial self-assembly for homogeneous nanoparticle monolayer enabled conformal “lift-on” thin film technology. Sci. Adv..

[68] Le Ru E.C., Etchegoin P.G. (2013). Quantifying SERS enhancements. MRS Bull..

[69] Neese F. (2012). The ORCA program system. Wiley Interdiscip. Rev. Comput. Mol. Sci..

[70] Neese F., Wennmohs F., Hansen A., Becker U. (2009). Efficient, approximate and parallel Hartree–Fock and hybrid DFT calculations. A ‘chain-of-spheres’ algorithm for the Hartree–Fock exchange. Chem. Phys..

[71] Neese F. (2003). An improvement of the resolution of the identity approximation for the formation of the Coulomb matrix. J. Comput. Chem..

[72] Takano Y., Houk K.N. (2004). Benchmarking the Conductor-like Polarizable Continuum Model (CPCM) for Aqueous Solvation Free Energies of Neutral and Ionic Organic Molecules. J. Chem. Theory Comput..

[73] Teixeira F., Cordeiro M.N.D.S. (2018). Improving Vibrational Mode Interpretation Using Bayesian Regression. J. Chem. Theory Comput..

[74] Teixeira F. (2017). VibAnalysis—Tools for Performing Vibrational Analysis on Molecular Systems. https://github.com/teixeirafilipe/vibAnalysis.

