# Supplementary material for: Self-Assembled Gold Nanoparticles as Reusable SERS Substrates for Polyphenolic Compound Detection
Source: Int J Mol Sci. 2024 Nov 28;25(23):12785. doi: 10.3390/ijms252312785 (PMC11640989; doi:10.3390/ijms252312785)
Supplement: Supplementary file 1 [file ijms-25-12785-s001.zip › ijms-3302403-Supplementary Information_rev3.pdf]

Supplementary Information

Self-Assembled Gold Nanoparticles as Reusable SERS Substrates for Polyphenolic Compound Detection

Arina Pavlova [1], Ksenia Maleeva [2], Ivan V. Moskalenko [1], Vadim Belyaev [1], Mikhail V. Zhukov [1], Demid Kirilenko [1], Kirill V. Bogdanov [2] and Evgeny Smirnov [1,*]

[1] Infochemistry Scientific Center, ITMO University, Lomonosova Str. 9, 191002 Saint Petersburg, Russia; arinapavlova@itmo.ru (A.P.); i.v.m.rostov.yar@gmail.com (I.V.M.); belyaev_ve@itmo.ru (V.B.); cloudjyk@yandex.ru (M.V.Z.); info.unifel@yandex.ru (D.K.)

[2] International Research and Educational Center for Physics of Nanostructures, ITMO University, Birzhevaya Liniya, 14, St. Petersburg, Russia; khnykina.kseniya@mail.ru (K.M.); kirw.bog@gmail.com (K.V.B.)

* Correspondence: evgeny.smirnov@alumni.epfl.ch

Table of Contents

Description	Page
**SI-1:** Descriptions of the ZIP-archive	S3
**SI-2:** Detailed information on AuNPs synthesis and preparation of enhancing substrates	S4
**SI-3:** Characterization of gold nanoparticles by UV/vis spectroscopy, zeta (ζ)-potential and dynamic light scattering measurements	S5
**SI-4:** Characterization of gold nanoparticles by transmission electron microscopy (TEM)	S8
**SI-5:** Characterization of gold nanoparticles energy dispersive X-Ray spectroscopy (EDX)	S9
**SI-6:** Raman spectra for pure silicon substrates covered with AuNPs	S10
**SI-7:** Comparison of experimental and DFT-calculated spectra for rhodamine 6G dye	S11
**SI-8:** Comparison of experimental and DFT-calculated spectra for chlorogenic acid dimer	S12

SI-1: Descriptions of the ZIP-archive

Archive S1. Studied structures in Cartesian coordinates, in particular, rhodamine 6G dye (RHOD6G.xyz), chlorogenic acid (CGA.xyz), dimer of chlorogenic acid (CGA_dimer.xyz).

SI-2: Detailed information on AuNPs synthesis and preparation of enhancing substrates

Synthesis of AuNPs

Modified Turkevich-Frens' method. In a nutshell, 27.67 mg of the solution containing 23.5 w% of gold was added to 100 mL of deionized water and then boiled in a round-bottom flask. Then, certain volume of 1 w% solution of reductant was rapidly injected into the flask. The latter varies the ratio of hydrogen tetrachloroaurate (Ш) to the reducing agent leading to the formation of nanoparticles of different diameters. Within 20 min of adding the reducing agent, the solution changed color from pale yellow to dark red, resulting in the formation of AuNPs.

Park's method or seed mediated growth. Briefly, a volume of 4 mL of a 20 mM aqueous $HAuCl_4 \cdot 3H_2O$ solution and 0.4 mL volume of a 10 mM $AgNO_3$ solution were added to 170 mL of deionized water. Then "seed nanoparticles" of small size obtained by Frens-Turkevich method were added to the solution. Subsequently, 30 ml of a 5.3 mM ascorbic acid solution was added slowly in a dropwise manner (~0.6 ml/min) to the resulting solution with constant stirring.

Preparation of enhancing substrates

MELLDs preparation. Briefly, 1 mM of tetrathiafulvalene (TTF) molecules dissolved in an organic phase (dichloroethane) were placed in contact with gold nanoparticle solution under vigorous stirring, emulsifying the mixture and leading to the formation of a continuous lustrous film of gold, so-called MELLD. Calculation of the number of nanoparticles required for one monolayer of close-packed nanoparticles in a hexagonal packing was carried out based on the average diameter and concentration obtained from the absorption spectra in according to the previously published data. (Smirnov, E. Assemblies of Gold Nanoparticles at Liquid-Liquid Interfaces; 2018. https://doi.org/10.1007/978-3-319-77914-0)

SI-3: Characterization of gold nanoparticles by UV/vis spectroscopy, zeta (ζ)-potential and dynamic light scattering measurements

In this Section the characterization of all synthesized AuNP colloidal solutions by UV-Vis spectroscopy and DLS methods are present. The obtained results for all synthesized samples are summarized in **Table S3-1** below.
The labeling of the samples should be read as the following ***Frens*** = nanoparticles synthesized by Frens method using one of reductant agent: ***Na_3Citr*** = sodium citrate, ***KAsc*** = potassium ascorbate or ***HAsc***= ascorbic acid; ***SMG*** = nanoparticles synthesized by Park method from 22 nm synthesized by Park method.

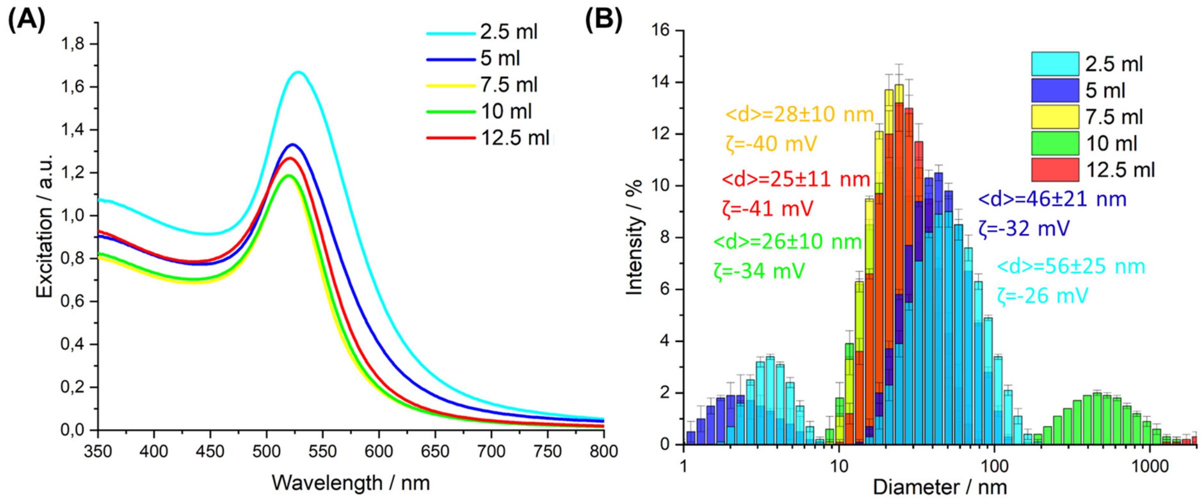

Figure S3-1. Characterization of gold nanoparticles synthesized by Frens-Turkevich method with trisodium citrate (samples ***Frens_Na_3Citr*** in the main text). UV-Vis spectra **(A)** and particles sized distribution with zeta-potentials accessed by DLS **(B)**.

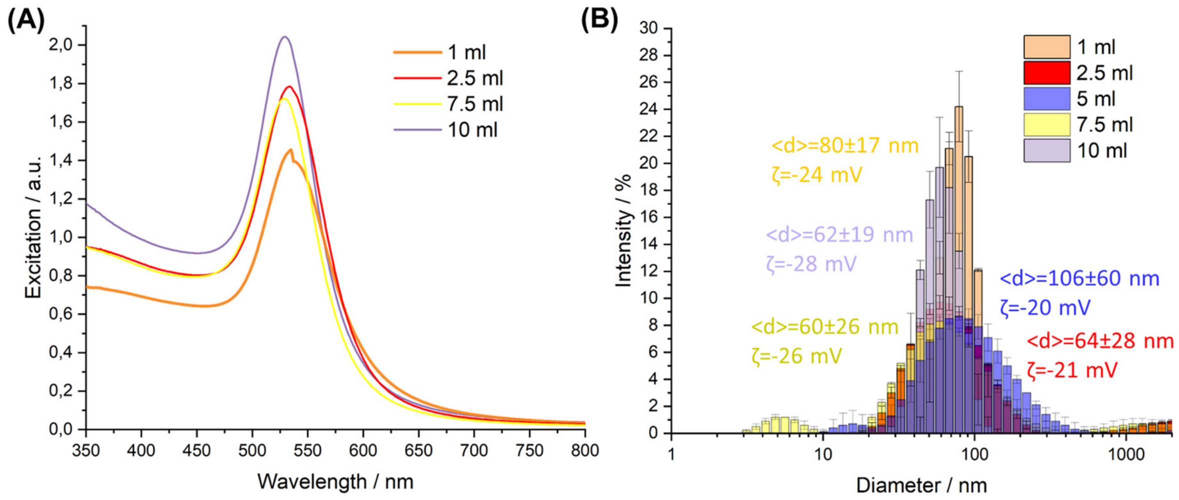

Figure S3-2. Characterization of gold nanoparticles synthesized by Frens-Turkevich method with ascorbic acid (samples ***Frens_HAsc*** in the main text). UV-Vis spectra **(A)** and particles sized distribution with zeta-potentials accessed by DLS **(B)**.

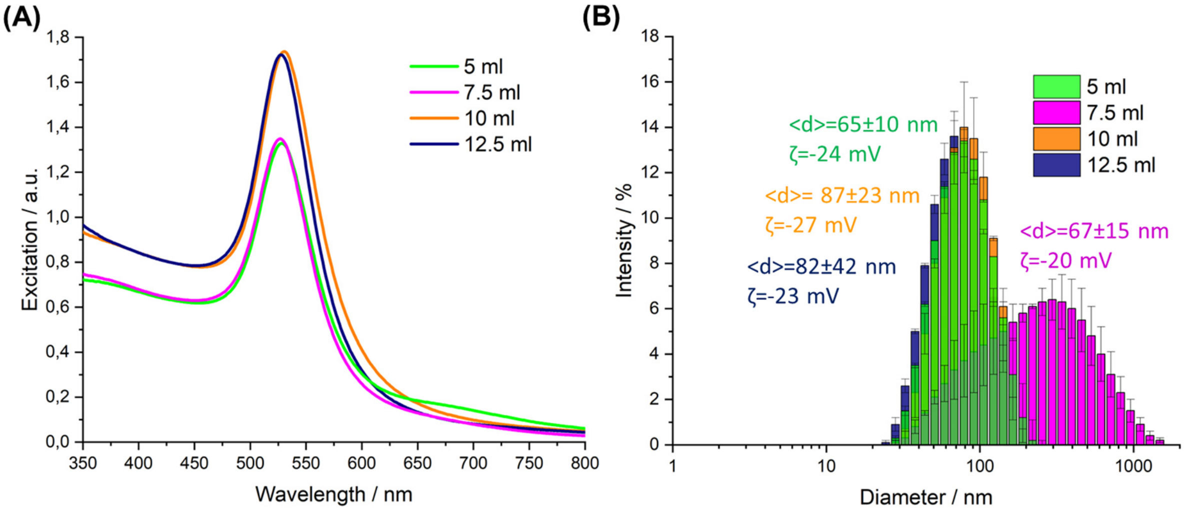

Figure S3-3. Characterization of gold nanoparticles synthesized by Frens-Turkevich method with potassium ascorbate (samples ***Frens_KAsc*** in the main text). UV-Vis spectra **(A)** and particles sized distribution with zeta-potentials accessed by DLS **(B)**.

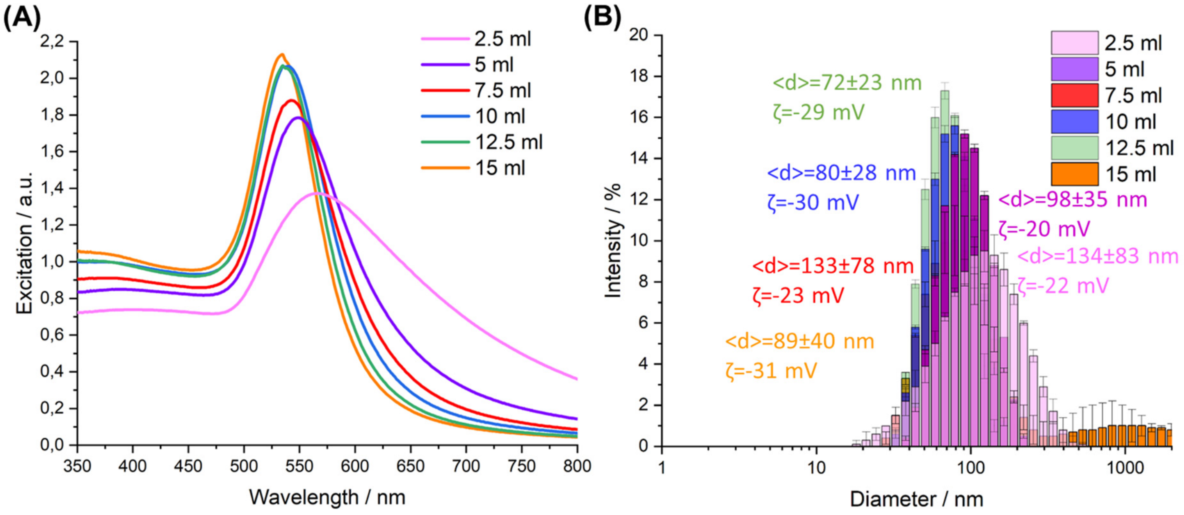

Figure S3-4. Characterization of gold nanoparticles synthesized by seed-mediated growth (samples ***SMG*** in the main text) from AuNPs synthesized by Frens-Turkevich method (*ca.* 22 nm). UV-Vis spectra **(A)** and particles sized distribution with zeta-potentials accessed by DLS **(B)**.

Table S3. Summary of UV–Vis spectroscopy and DLS characterization of synthesized AuNPs by different methods.

Synthesis method	$V_{reductant}$ or $V_{seed\ NPs}$ / ml	$<d_{UV\text{-}Vis}>$ / nm	$C_{UV\text{-}Vis}$ / part·µl–1	$<d_{DLS}>$ / nm	$<\zeta>$ / mV
Frens Trisodium citrate (**Frens_Na3Citr**)	2.5	44	$8.33\cdot10^{7}$	56±25	–26
	5	19	$1.02\cdot10^{9}$	46±21	–32
	7.5	19	$9.12\cdot10^{8}$	28±10	–40
	10	16	$1.72\cdot10^{9}$	26±10	–34
	12.5	14	$2.73\cdot10^{9}$	25±11	–41
Frens Potassium ascorbate (**Frens_KAsc**)	1	58	$4.98\cdot10^{7}$	80±17	–24
	2.5	54	$3.86\cdot10^{7}$	64±28	–21
	5	56	$2.81\cdot10^{7}$	106±60	–20
	7.5	41	$9.13\cdot10^{7}$	60±26	–26
	10	44	$8.35\cdot10^{7}$	62±19	–28
Frens Ascorbic acid (**Frens_HAsc**)	5	41	$7.05\cdot10^{7}$	65±10	–24
	7.5	39	$8.41\cdot10^{7}$	67±15	–20
	10	47	$5.81\cdot10^{7}$	87±23	–27
	12.5	39	$1.05\cdot10^{8}$	82±42	–23
Seed-Mediated Growth from 22 nm seed NPs (**SMG**)	2.5	99	$6.95\cdot10^{6}$	134±83	–22
	5	80	$1.28\cdot10^{7}$	98±35	–20
	7.5	72	$1.78\cdot10^{7}$	133±78	–23
	10	67	$2.36\cdot10^{7}$	80±28	–30
	12.5	58	$3.57\cdot10^{7}$	72±23	–29
	15	56	$4.11\cdot10^{7}$	89±40	–31

SI-4: Characterization of gold nanoparticles by transmission electron microscopy (TEM)

In this Section, the characterization of all synthesized AuNP colloidal solutions by TEM microscopy is present. The mean diameters for synthesized AuNPs were calculated based on analysis of tens individual AuNPs (typically, 30 to 40 individual nanoparticles), standard deviation shows the calculated diameter variations.

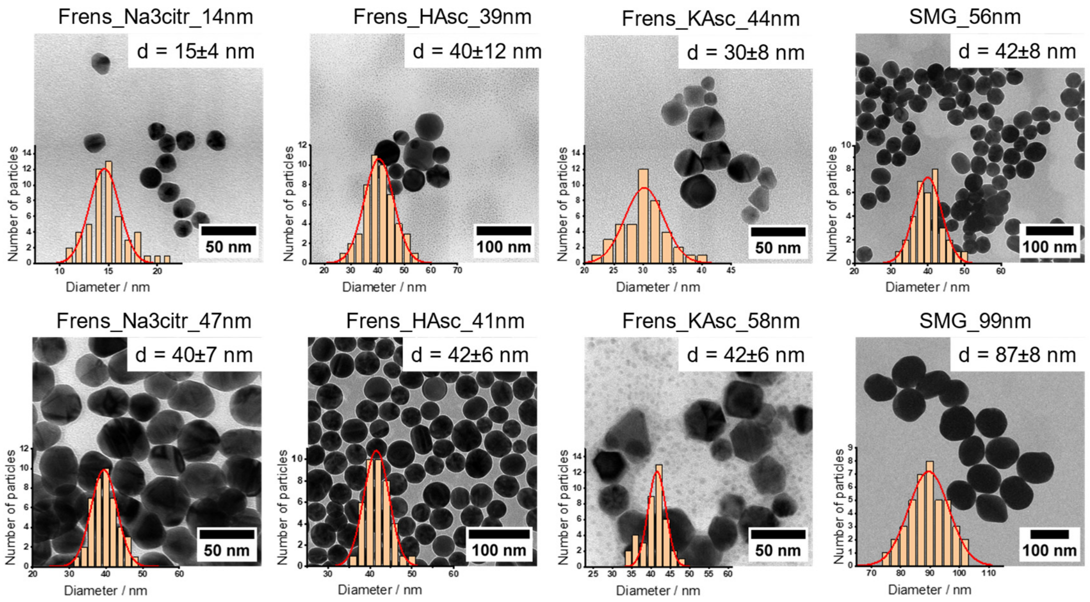

Figure S4. TEM-images of gold nanoparticles synthesized by Frens-Turkevich with different reducing agents (*Frens*) and seed-mediated growth (*SMG*) methods and their mean diameters with standard deviation. Insets: Bar-diagram of particles size distribution based on TEM-images data processing of 30 to 40 individual AuNPs (red curve is Gaussian fit).

SI-5: Characterization of gold nanoparticles energy dispersive X-Ray spectroscopy (EDX)

In this Section, the characterization of the substrate made of 72 nm AuNPs (seed-mediated growth method) by EDS mapping is present. Three maps were recorded namely Si Kα at 1.74 keV, S Kα at 2.31 keV (as TTF may stack on the surface of gold nanoparticles) and Au Mα at 2.12 keV. As expected, due to penetration depth, which is higher than the size of Au nanoparticles, the EDX map of silicon based on its K_α-line shows the flat background. At the same time, the EDX map of gold based on its M_α-line demonstrates features that can be also seen on SE-image such as tiny cracks, pin-holes *etc.* The presence of sulfur is negligible according to its EDX mapping.

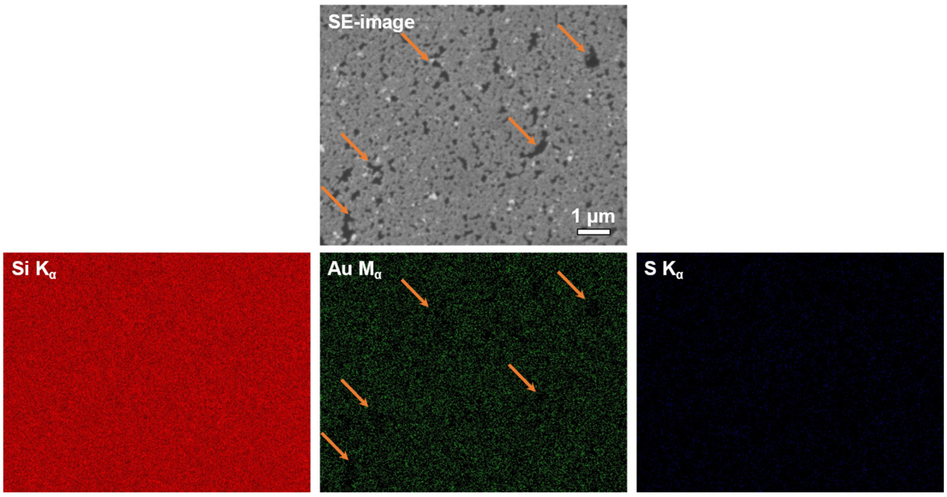

Figure S5. EDS mapping of the prepared silicon substrate covered by AuNPs: top panel is SE-image of the substrate with marked tiny cracks, pin-holes: bottom panels are EDS-maps for Si Kα at 1.74 keV, Au Mα at 2.12 keV and S Kα at 2.31 keV (as TTF may stack on the surface of gold nanoparticles), respectively, from left to right.

SI-6: Raman spectra for pure silicon substrates covered with AuNPs

The Figure S6 presents Raman spectra for all synthesized AuNPs deposited on silicon substrates. It demonstrates the absence of any additional band corresponding to TTF or other pollutant on the surface after plasma cleaning before the benchmarking experiment.

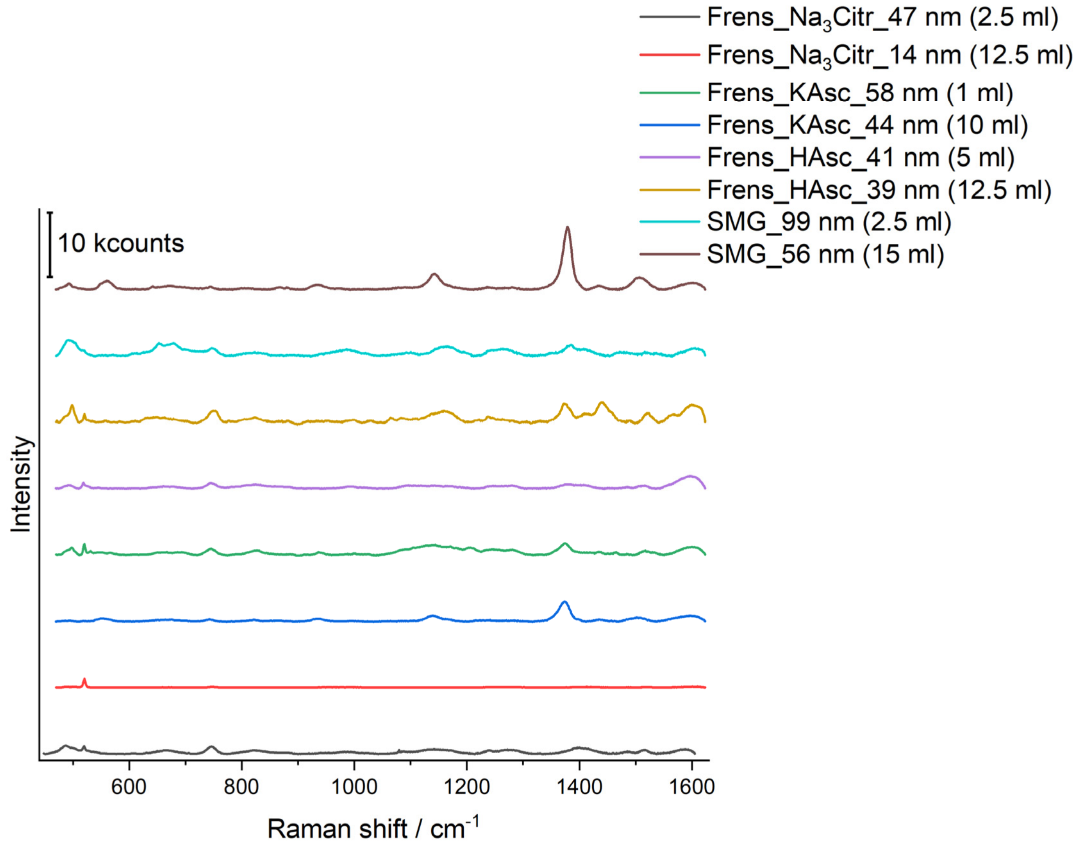

Figure S6. Raman spectra for all synthesized AuNPs deposited on silicon substrates. The labeling of the samples should be read as the following Frens = nanoparticles synthesized by Frens-Turkevich method using one of reductant agent: Na3Citr = sodium citrate, KAsc = potassium ascorbate or HAsc= ascorbic acid; SMG = nanoparticles synthesized by Park method.

SI-7: Comparison of experimental and DFT-calculated spectra for rhodamine 6G dye

The Figure S7 represents a comparison of the experimentally recorded and DFT-calculated Raman spectra for rhodamine 6G dye deposited on a clean silicon substrate. The best match between the theoretical and experimental Raman spectra for R6G can be achieved with the scaling factor for Raman shift of 0.96.

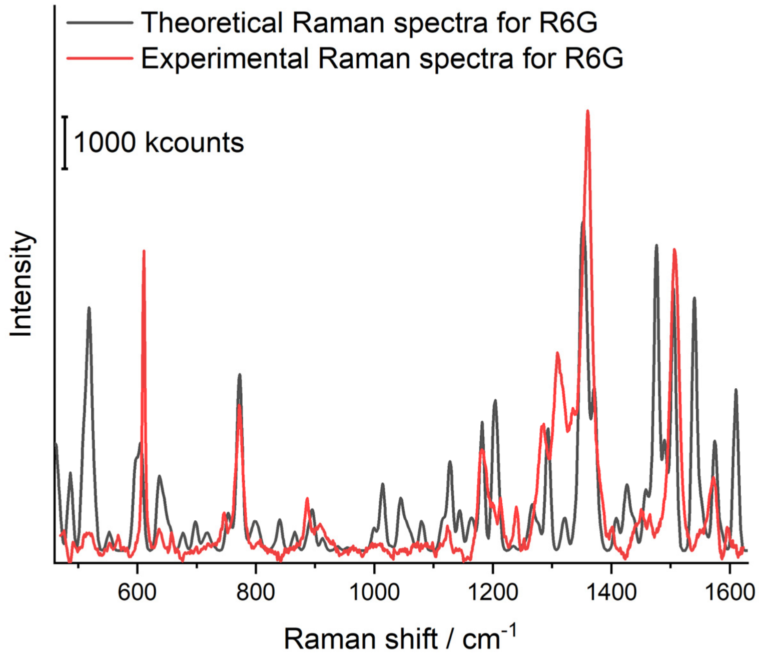

Figure S7. Comparison of the experimental and DFT-calculated Raman spectra for rhodamine 6G dye deposited on a silicon substrate.

SI-8: Comparison of experimental and DFT-calculated spectra for chlorogenic acid dimer

Table S8 contains observed vibrations bands for CGA dimer both calculated through DFT and experimentally recorded.

Table S8. Summary of experimentally observed (exp) and DFT-calculated (theor) both scaled and unscaled Raman bands with the following assignments to the vibrational bands of CGA dimer.

Observed Raman bands ν_{633}^{exp} / cm^{-1}	Theoretical Raman bands ν_{633}^{theor} / cm^{-1}		Assignment of the vibrational band
	Unscaled	Scaled	
655	677.1	656.7	Cycl [ν(C–C)]
679	697.4	676.5	Phe [ν(C=C)]
728	750.2	727.7	Cycl [ν(C=O) + δ(O–C–O)]
758	777.8	754.4	Phe [δ(C–C=C)]
788	830.0	788.5	Phe [δ(C–C=C)]
808	850.9	807.5	Cycl [ν(C–O)]
859	885.1	858.5	Ester [ν(C=O)]
899	924.1	896.3	Phe [τ (C–C=C–H) + τ (C–C=C–C)]
918	946.6	918.2	Phe [ν(C–H)]
859	885.1	858.5	Ester [ν(C=O)]
899	924.1	896.3	Phe [τ (C–C=C–H) + τ (C–C=C–C)]
919	946.6	918.2	Phe [ν(C–H)]
953	1000.9	950.9	Cycl [ν(C–O) + ν (C–C) + δ(C–C–C)]
974	1022.2	971	Cycl [ν(C–O) + ν(C–C)]
992	1032.6	991.6	Cycl[ν(C–O) + ν(C–C) + ν(C–O)]
1013	1044.6	1013.2	Cycl[ν(C–C)]
1070	1096.9	1064.0	Cycl[ν(C–C)]
1158	1191.3	1155.5	Cycl[ν(C–C) + ν(C–O)]

1200	1240.6	1203.4	Cycl[ν(C–C) + ν(H–O)]
1275	1328.6	1275	Phe [ν(C–H)]
1294	1347.3	1293.4	Cycl[ν(C–C)]
1321	1376.6	1322	Phe [ν(C=C)]
1340	1393.9	1338	Phe [ν(C=C)]
1358	1412.9	1356.4	Cycl[ν(C–C)]
1376	1432.7	1375	Phe [ν(C=C)]
1394	1442.4	1399.1	Cycl[δ(C–O–H) + ν(C–C) + δ(C–C–H + δ(H–C–O)]
1420	1465.0	1421.1	Cycl[δ(H–C–H) + δ(C–O–H)]
1442	–	–	N/A
1490	–	–	N/A
1532	–	–	N/A
1561	–	–	N/A
1587	–	–	N/A
1615	1665.3	1615.3	Phe [ν(C=C)]

Types of vibration: ν. stretching; δ. bending; τ. torsion. *Abbreviations:* Phe – phenyl group; Ester – ester group; Cycl. – cyclohexane group (quinic acid).
